# Igf Signaling is Required for Cardiomyocyte Proliferation during Zebrafish Heart Development and Regeneration

**DOI:** 10.1371/journal.pone.0067266

**Published:** 2013-06-26

**Authors:** Ying Huang, Michael R. Harrison, Arthela Osorio, Jieun Kim, Aaron Baugh, Cunming Duan, Henry M. Sucov, Ching-Ling Lien

**Affiliations:** 1 Heart Institute, The Saban Research Institute of Children’s Hospital Los Angeles, Los Angeles, California, United States of America; 2 Program of Developmental Biology and Regenerative Medicine, The Saban Research Institute of Children’s Hospital Los Angeles, Los Angeles, California, United States of America; 3 Craniofacial Biology Graduate Program, Ostrow School of Dentistry, University of Southern California, Los Angeles, California, United States of America; 4 Department of Molecular, Cellular and Developmental Biology, University of Michigan, Ann Arbor, Michigan, United States of America; 5 Broad CIRM Center for Regenerative Medicine and Stem Cell Research, University of Southern California, Los Angeles, California, United States of America; 6 Department of Surgery, Keck School of Medicine, University of Southern California, Los Angeles, United States of America; 7 Department of Biochemistry & Molecular Biology, Keck School of Medicine, University of Southern California, Los Angeles, United States of America; Institute of Clinical Medicine, National Cheng Kung University, Taiwan

## Abstract

Unlike its mammalian counterpart, the adult zebrafish heart is able to fully regenerate after severe injury. One of the most important events during the regeneration process is cardiomyocyte proliferation, which results in the replacement of lost myocardium. Growth factors that induce cardiomyocyte proliferation during zebrafish heart regeneration remain to be identified. Signaling pathways important for heart development might be reutilized during heart regeneration. IGF2 was recently shown to be important for cardiomyocyte proliferation and heart growth during mid-gestation heart development in mice, although its role in heart regeneration is unknown. We found that expression of *igf2b* was upregulated during zebrafish heart regeneration. Following resection of the ventricle apex, *igf2b* expression was detected in the wound, endocardium and epicardium at a time that coincides with cardiomyocyte proliferation. Transgenic zebrafish embryos expressing a dominant negative form of Igf1 receptor (dn-Igf1r) had fewer cardiomyocytes and impaired heart development, as did embryos treated with an Igf1r inhibitor. Moreover, inhibition of Igf1r signaling blocked cardiomyocyte proliferation during heart development and regeneration. We found that Igf signaling is required for a subpopulation of cardiomyocytes marked by *gata4:EGFP* to contribute to the regenerating area. Our findings suggest that Igf signaling is important for heart development and myocardial regeneration in zebrafish.

## Introduction

Most adult cardiomyocytes in mammals are generally thought to have permanently exited the cell cycle and are thus unable to proliferate [1], [2], [3], [4]. As a result, adult mammalian hearts fail to regenerate in response to damage or disease. This fundamental problem can lead to heart failure following myocardial infarction, which remains the leading cause of death in developed countries [5]. Therefore, regenerative therapeutics are desperately needed for patients with coronary heart diseases. More recent evidence suggests that mammalian cardiomyocytes can also undergo limited proliferation for homeostatic renewal and after myocardial infarctions [6], [7]. However, the number of proliferating cardiomyocytes is very small and the natural turnover of adult cardiomyocytes is not sufficient for regenerating damaged hearts. Sereval attempts have been undertaken to identify mitogens of mouse cardiomyocytes and promoting cardiomyocyte proliferation by addition of growth factors to mouse hearts has been shown to enhance the repair process in these models [8], [9].

Due to its natural capability to regenerate the heart and other organs, the zebrafish can be used as a blueprint for the design of regenerative therapies. Zebrafish hearts fully regenerate within 1–2 months after amputation of 20% of the ventricle [10], [11], [12], [13], [14], [15]. Zebrafish hearts regenerate by undergoing dedifferentiation and proliferation of cardiomyocytes [16], [17]. Cardiomyocyte proliferation starts from 7 days post amputation (dpa) and peaks at 14 dpa [10]. Recent findings from Kikuchi et al. suggest that a population of cardiomyocytes marked by *gata4:EGFP* in the sub-epicardial compact myocardium plays a major role in replacing lost cardiomyocytes [17]. The signals that induce cardiomyocyte proliferation have not been identified. Since *gata4:EGFP* positive cardiomyocytes are mainly located in compact myocardium, it was postulated that the mitogens for this population likely originate from the adjacent epicardium [14].

Zebrafish heart regeneration recapitulates many steps that occur during embryonic development. During heart growth of mid-gestation mouse embryos, mitogens originating from the epicardium play essential roles in inducing cardiomyocyte proliferation in the myocardium. Insulin like growth factor (IGF) 2 was recently identified as one of these mitogens. Its expression is regulated by hepatic erythropoietin (EPO) and indirectly by retinoic acid (RA) [18], [19]. IGF2 is a ligand for both IGF1 receptor (IGF1R) and insulin receptor (INSR) isoform A and signals through several signaling cascades including the Akt-PI3K and RAF-MEK-ERK1/2 pathway [20], [21]. However, IGF2 is probably not the only epicardial mitogen for cardiomyocytes, since *Igf2* knockout mice still have 20–25% cardiomyocytes undergoing proliferation [19]. In zebrafish, there are two *igf2* genes, *igf2a* and *igf2b* [22]. Igf signaling has been shown to be important for zebrafish fin regeneration, and specifically for signaling between the blastema and wound epidermis [23].

In this study, we determined the functions of Igf signaling in zebrafish heart regeneration. We found that *igf2b* is expressed in the epicardium, wound, and endocardium. Blocking Igf signaling using a dominant-negative form of Igf1 receptor (dn-Igf1r) or a selective Igf1r inhibitor results in impaired heart development in zebrafish. We further demonstrated that Igf signaling is required for cardiomyocyte proliferation during zebrafish heart development and regeneration. Specifically, the contribution of the *gata4:EGFP* population to the regenerating area is impaired. Our work identifies an evolutionarily conserved role of IGF signaling in heart development and an important role in cardiomyocyte proliferation during heart regeneration.

## Methods

### Zebrafish Usage and Experimental Manipulation

Zebrafish used in this study were maintained using standard methods [24]. The transgenic lines *Tg*(*hsp70:dnigf1ra-GFP*), *Tg(myl7:nDsRed)* (previously named *cmlc2:nDsRed*), *Tg(myl7:EGFP)* (previously named *cmlc2:EGFP*) and *Tg(gata4:EGFP)* have been described before [25], [26], [27], [28]. Fish that were older than 6 months were used for regeneration studies. The heart amputation procedure was performed as described [10], [29], [30], [31]; all protocols were approved by Children’s Hospital Los Angeles IACUC. For transgene induction, adult zebrafish and control zebrafish were heat shocked once daily by transferring fish tanks into a 38°C water bath for 1 h. To induce transgene expression during heart development, *Tg*(*hsp70:dnigf1ra-GFP*) and control sibling embryos were heated shocked at 40°C for 30 min. For pharmacological inhibition of IGF, adult transgenic fish or wildtype *Ekk* fish were treated with Igf1r inhibitor NVP-AEW-541 (Cayman Chemical) at 5 µM and embryos were treated in E3 media containing the same inhibitor at 10 µM. DMSO was used as a control in both embryo and adult experiments. To measure cardiomyocyte proliferation, BrdU was added in to fish water from 7dpa to 10dpa at 50 µg/mL for in vivo labeling of adult fish; and from 48–72 hpf at 5 mg/mL for labeling of embryos [32].

### *In situ* Hybridization (ISH)

ISH was performed as described [31], [33]. The probes for *igf2a* and *igf2b* were generated by PCR using the following primers: *igf2a*: F-CCATCCCAAATGGCAACAAACAA, R-TCCACAGCCAGCCAACATTTTC; *igf2b*: F-AAAGACAGGATCGTTTGCAC, R-CTGGAACAGGAATCTAATTTTAC.

### Quantitative RT-PCR

Total RNA was extracted using TRIzol® RNA isolation reagents from Invitrogen. cDNA was synthesized using SuperScript® III First-Strand synthesis kits (Invitrogen). Real-time quantitative PCR was carried out using the Roche LightCycler® 480 Real-Time PCR System. RT-PCR reactions were carried out in a 20 µl volume with 0.2 µM gene specific primers and detected with a universal probe from Roche. Zebrafish *eef1a1l1* primer was used as an internal control to ensure cDNA quantity and integrity. Cycle values were collected in triplicate at each time point. *eef1a1l1*-probe#73: F-CCTCTTTCTGTTACCTGGCAA, R-CTTTTCCTTTCCCATGATTGA; *igf2b*-probe#110: F-AGCTGGTGGACGCTCTACA, R-GAGAACGTCGACTGTTTGACC.

### Immunostaining

Immunostaining was carried out as described [31]. Briefly, wild type embryos or adult hearts were harvested at 76 hpf and 10 or 14dpa, respectively. They were then fixed, sectioned and stained with anti-BrdU antibody (Abcam) and anti-MEF2 (Santa Cruz) antibody. Both were used at a 1∶500 dilution after heat antigen retrieval.

### Imaging of Zebrafish Embryos and Adult Hearts

Imaging of embryos and sections was performed using confocal microscopy (Zeiss LSM710 710) and a fluorescent microscope (Leica DMIRE2). Images were acquired as a lambda stack and processed using Zen (Zeiss, Germany), then quantified using Image J (NIH). Whole mount confocal imaging was used to image hearts from *Tg(gata4:EGFP)* fish and *Tg(myl7:ndsRed; hsp70:dnigf1ra-GFP)* embryos. After removal from the zebrafish, hearts were cleaned in 1x PBS then fixed briefly (approximately 30 seconds) in 4% PFA in PBS. Hearts or embryos were then mounted and orientated in 1% low melting-point agarose to be imaged using an inverted confocal microscope.

### Quantification and Statistics

To quantify cardiomyocyte proliferation in embryos, all visible sections of hearts from each embryo were imaged using a 20X objective. The numbers of Mef2^+^ and Mef2^+^BrdU^+^ cells were manually counted using Image J software (NIH). The percentages of Mef2^+^BrdU^+^ cells from each embryo were averaged to determine the BrdU incorporation rate.

To quantify cardiomyocyte proliferation in adult hearts, three sections showing the largest wounds were selected from each heart, and images were taken using a 20X objective. Using Image J, the numbers of Mef2^+^ and Mef2^+^BrdU^+^ cells were manually counted within a defined region (yellow box shown in figures) including the majority of Mef2^+^BrdU^+^ cardiomyocytes near and within the injured area. The percentages of Mef2^+^BrdU^+^ cells from the three selected sections were averaged to determine a proliferation rate for each heart.

To quantify *gata4:EGFP* positive cardiomyocyte proliferation in adult hearts, three sections showing the largest wounds were selected from each heart, and images were taken using a 10X objective. Using Image J, GFP^+^BrdU^+^ cells were manually counted and the GFP-expressing area was measured. GFP^+^BrdU^+^ double positive cells per unit area from the three selected sections were averaged to determine a proliferation rate for each heart.

To quantify scar formation after DMSO or Igf1r inhibitor treatment at 30 dpa in adult hearts, three sections showing the largest scar area were selected from each heart. Using Image J, the area of scar and ventricle was measured. The ratio of scar/ventricular area from the three selected sections was averaged for each heart.

Student's t-test was used to determine statistical significance (p<0.05).

### AFOG Staining

To identify collagen scars, AFOG staining was performed as described [10].

## Results

### *igf2b* is Up-regulated during Zebrafish Heart Regeneration

**Figure 1:**
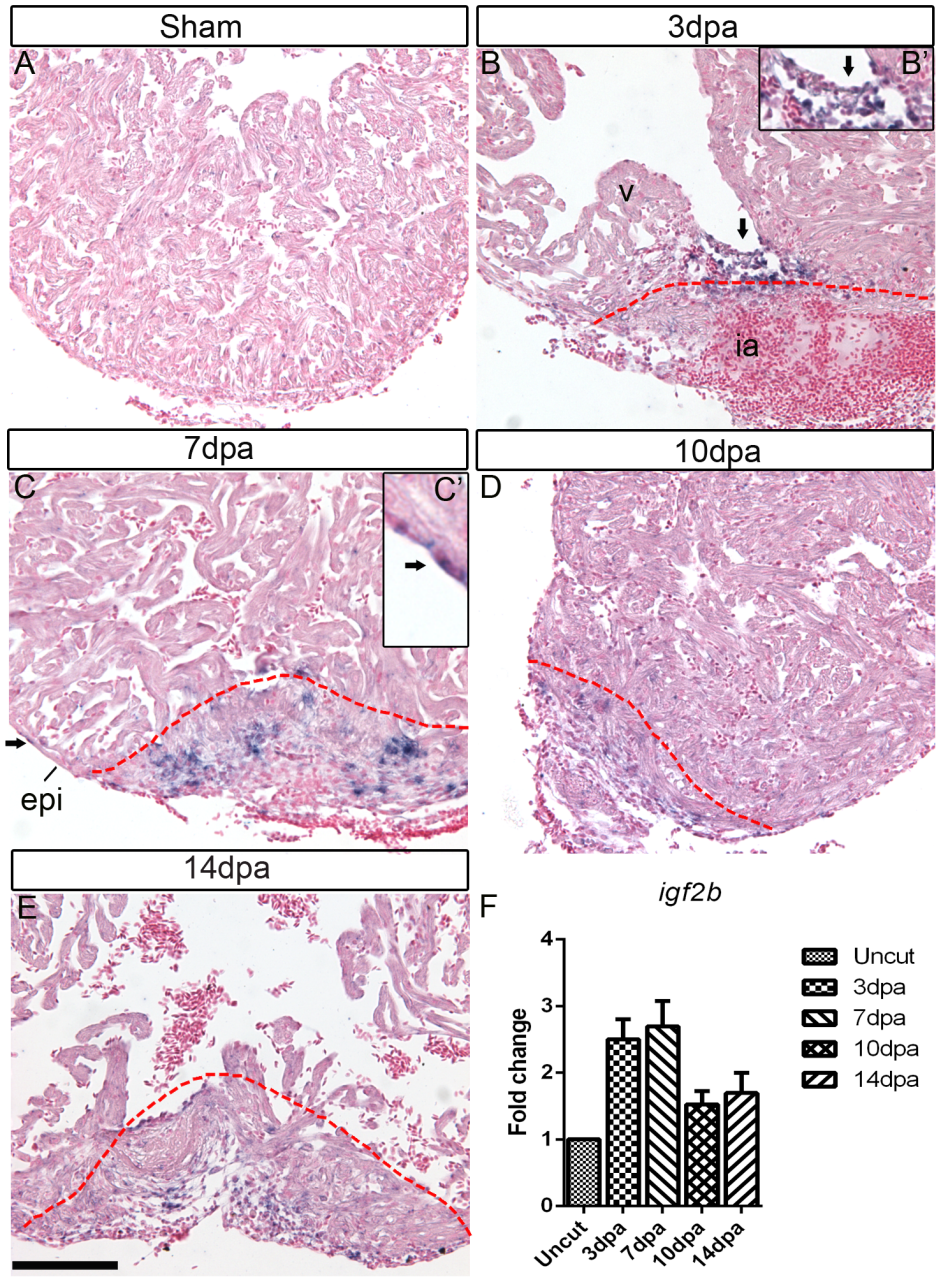
*igf2b* is upregulated during zebrafish heart regeneration. *In situ* hybridization (ISH) was performed on sham operated hearts (A) and 3 dpa (B), 7 dpa (C), 10 dpa (D) and 14 dpa (E) regenerating hearts. There is no detectable *igf2b* expression in sham-operated hearts by ISH, whereas strong expression is induced in endocardium (arrows in B and inset B’) and epicardium (arrows in C and inset C’) near the wound following amputation of the ventricular apex. Red dashed lines mark the amputation site and wound area. v: ventricle, ia: injured area, epi: epicardium. Scale bar = 100 µm. (F) Quantitative RT-PCR of *igf 2b* expression at 3, 7, 10 and 14 dpa compared to uncut hearts.

Using microarray based gene expression analysis, we previously identified *igf2* as one of the candidate growth factor genes that are upregulated during zebrafish heart regeneration [31]. To determine the spatial expression patterns of *igf2* after ventricular resection, we performed section *in situ* hybridization (ISH) of *igf2a* and *igf2b* in wild type zebrafish hearts. In sham-operated hearts, there was no detectable *igf2a* or *igf2b* expression (Fig. 1A, Fig. S1A). Between 3–10 days post-amputation (dpa), *igf2b* was expressed in part of the endocardium close to the wound, in epicardium adjacent to the amputation plane, and in the wound (Fig. 1B–E). Its expression starts to decrease by 10 dpa (Fig. 1D–F). This is consistent with a functional role of Igf signaling in regeneration, as the peak of cardiomyocyte proliferation occurs between 7–14 dpa [10]. Interestingly, in mice *Igf2* is expressed in both the epicardium and endocardium of the developing hearts [19]; similarly, *igf2b* is expressed in the zebrafish embryonic hearts [34]. Induction of *igf2b* expression during zebrafish heart regeneration suggests that developmental genes are reactivated during the regenerative process. We did not detect *igf2a* expression in the regenerating hearts using ISH (Fig. S1).

### Zebrafish Embryonic Heart Development Requires Igf Signaling

**Figure 2:**
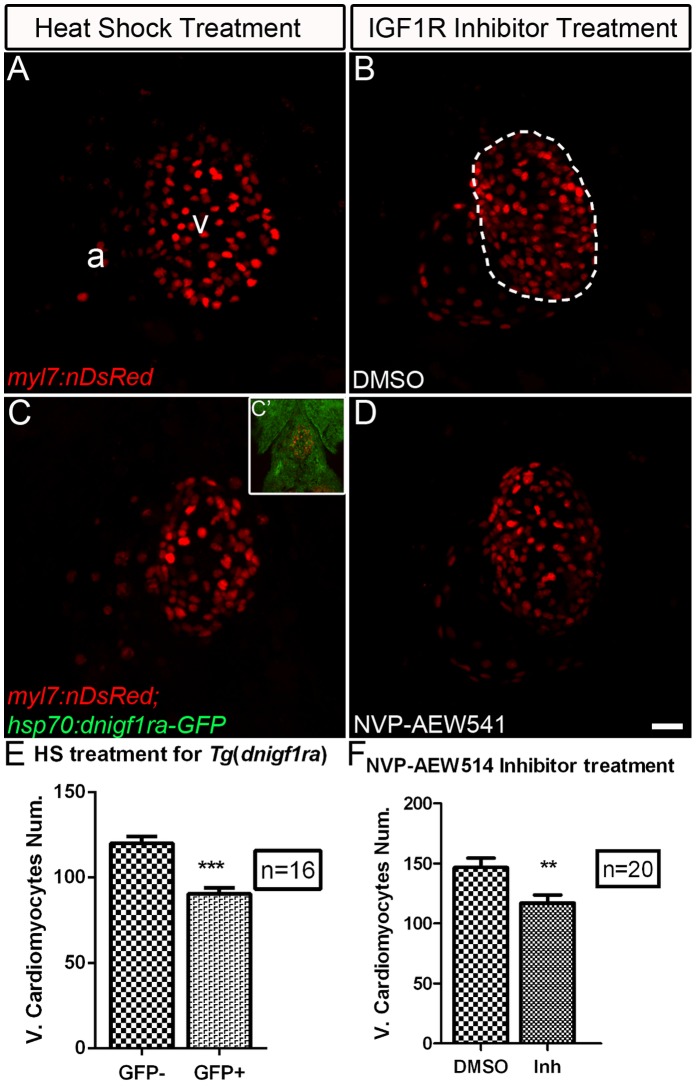
Igf signaling is required for proper cardiomyocyte number in zebrafish embryonic hearts. The number of ventricular cardiomyocytes (area encircled by dotted line) number decreased in embryos when Igf1r signaling was blocked. (A) *Tg(myl7:nDsRed)* single transgenic and (C) *Tg(hsp:dnigf1ra-GFP; myl7:nDsRed)* double transgenic embryos (n = 16) were heat shocked at 40°C for 30 min around 48 and 72 hpf and observed at 76 hpf. *dnigf1ra* embryos can be identified by GFP expression (C’ inset). *Tg(myl7:nDsRed)* single transgenic siblings (n = 16) were used as controls. *myl7:nDsRed* embryos were treated with Igf1r inhibitor (D) (n = 20) and DMSO as a control (B) (n = 20) from 48 to 72 hpf and observed at 76 hpf. a: atrium, v: ventricle. Scale bar = 20 µm. (E, F) Quantification of mean ventricular cardiomyocyte number ± S.E. (***p<0.0001; **p<0.001). Fewer ventricular cardiomyocytes were observed around 76 hpf after Igf1r signaling was blocked.

Embryos in which *igf2b* activity had been knocked down using morpholino oligonucleotide injection showed early developmental heart defects in atrioventricular boundary specification and cardiac looping [34]. IGF2 signaling was recently shown to play an important role in cardiomyocyte proliferation during mid-gestation cardiac growth in mice [19]. A role for Igf signaling in heart growth has not been previously studied in fish embryos. To determine the role of Igf signaling in zebrafish heart development at a later stage, we utilized an inducible transgenic zebrafish line in which a dominant-negative Igf1ra variant fused to GFP is driven by a heat shock promoter [*Tg*(*hsp70:dnigf1ra-GFP*)] [25]. *Tg*(*hsp70:dnigf1ra-GFP*) fish were crossed to *Tg(myl7:nDsRed)* fish, in which cardiomyocytes are marked with red fluorescence [26]; embryos were heat shocked at 40°C for 30 min at 48 hr and 72 hr post-fertilization, and ventricular cardiomyocytes were counted at 76 hr. We measured a 24.6% decrease (mean ± SEM: GFP^−^ = 120.1±3.86; GFP^+^ = 90.5±3.49) in cardiomyocyte number in double transgenic embryos relative to heat shocked sibling control embryos (Fig. 2A, C, and E). We further tested a selective Igf1r chemical inhibitor, NVP-AEW-541, which blocks the ATP binding site of mammalian IGF1R [35]. NVP-AEW-541 is also effective in blocking Igf1r during zebrafish development and was shown to inhibit fin regeneration in adult fish [23], [25]. Igf1r inhibitor-treated zebrafish embryos have 20.2% fewer cardiomyocytes (mean±SEM: DMSO = 146.6±7.80; NVP-AEW541 = 117.0±16.70) compared to DMSO treated embryos (Fig. 2B, D, and F; see also Fig. S2A–E). We also observed abnormal heart looping (Fig. S3A and B) and reduced blood circulation in NVP-AEW-541 treated embryos, similar to previously reported defects in embryos injected with *dnigf1r* RNA [34], further validating the efficacy of NVP-AEW-541.

**Figure 3:**
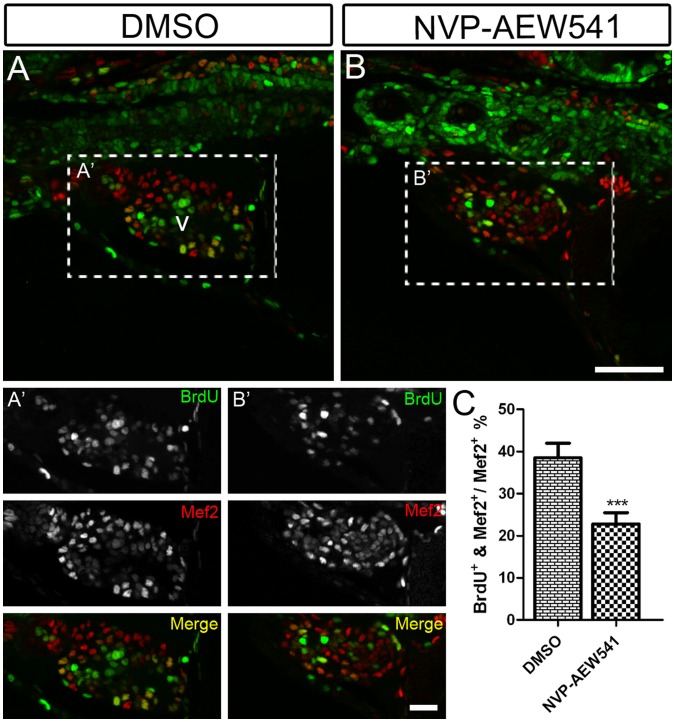
Igf signaling is required for cardiomyocyte proliferation during zebrafish heart development. Wild type fish were treated with DMSO as a control (n = 5) (A and A’) and the Igf1r inhibitor NVP-AEW541 (n = 7) (B and B’) from 48–72 hpf. BrdU was added for the same time period. BrdU (green) and Mef2 (red) double positive cells indicate proliferating cardiomyocytes (A, A’, B, B’). A’ and B’ are images of the dashed boxes in A and B. (A’ and B’), BrdU (green) and Mef2 (red) staining were shown as black and white or merged color images. Scale bar: (B) = 50 µm, (B’) = 20 µm. v: ventricle. (C) Quantification of BrdU positive cardiomyocytes (Mef2 positive) ± S. E. A significant decrease (***p<0.0001) in cardiomyocyte proliferation was detected in embryos treated with NVP-AEW541.

To further confirm the defects we observed after blocking Igf signaling were due to defects in cardiomyocyte proliferation, we measured DNA synthesis using BrdU incorporation from 48–72 hpf. Cardiomyocytes were identified by Mef2 staining. We observed a 40.79% decrease (mean ± SEM: DMSO = 38.51±3.43%; NVP-AEW541 = 22.80±2.66%) in BrdU incorporation (Fig. 3C) in NVP-AEW-541 treated embryos (Fig. 3B and B’), compared to control embryos treated with DMSO (Fig. 3A and A’). These results suggest that Igf signaling is required for cardiomyocyte proliferation during heart development.

### Igf Signaling is Necessary for Cardiomyocyte Proliferation During Zebrafish Heart Regeneration

**Figure 4:**
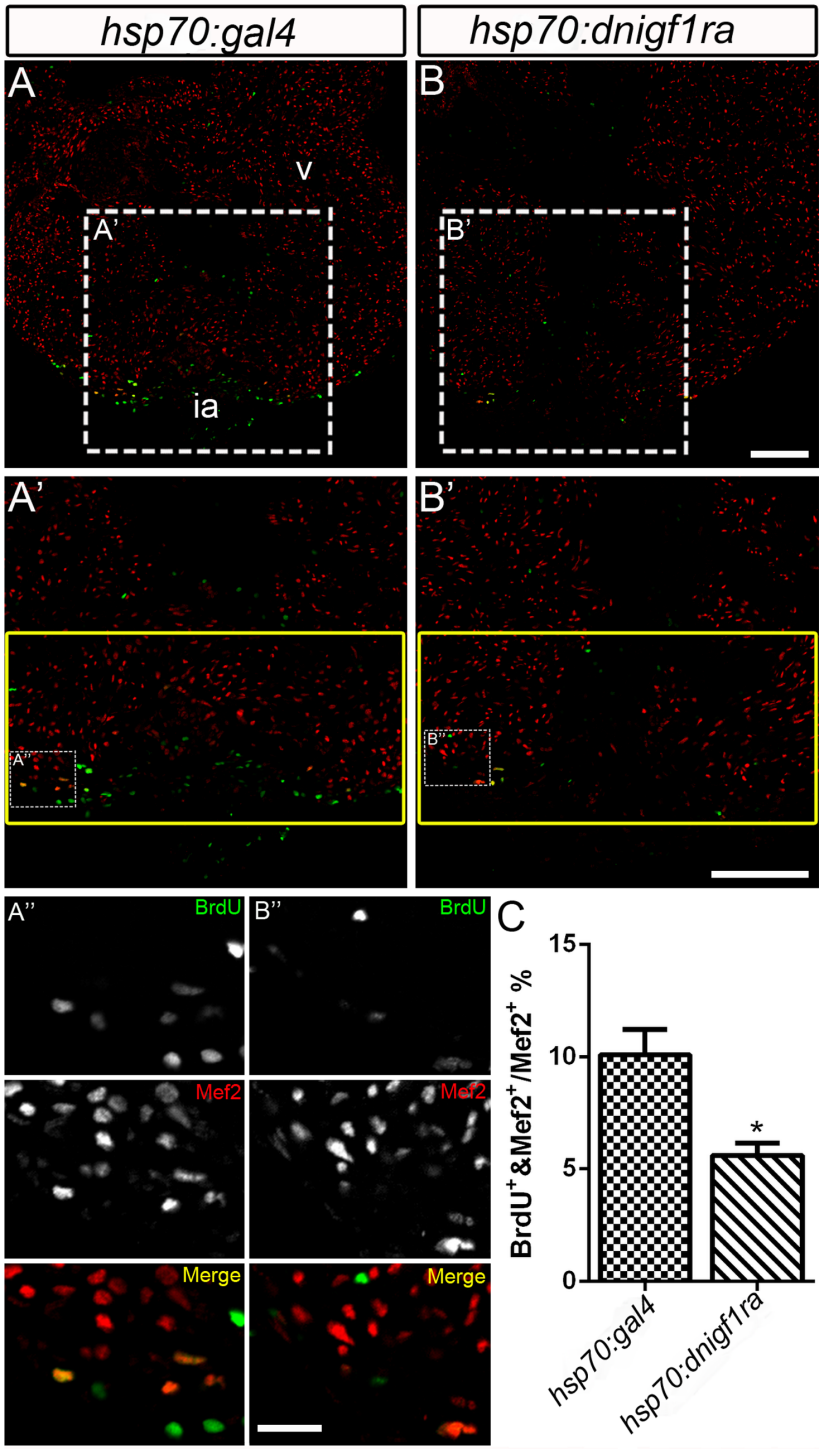
Igf signaling is required for cardiomyocyte proliferation during zebrafish heart regeneration. Cardiomyocyte proliferation was decreased after Igf signaling was blocked during zebrafish heart regeneration. *Tg(hsp70:gal4)* control (n = 5) (A, A’, A’’) and *Tg(hsp70:dnigf1ra-GFP)* transgenic zebrafish (n = 7) (B, B’,B’’) zebrafish were heat shocked for 1 h at 38°C after amputation from 2–10 dpa. BrdU (green) and Mef2 (red) double positive cells indicate proliferating cardiomyocytes (A, A’, B, B’). A’ and B’ are the higher magnification images of the dashed boxes in A and B. A’’ and B’’ are the higher magnification images of the dashed boxes in A’ and B’. The yellow box indicates the wound area; cardiomyocytes were counted in this region. (A’’ and B’’), BrdU (green) and Mef2 (red) staining were shown as separated channel images (black and white) or merged color images. ia: injured area, v: ventricle. Scale Bar: (B, B’) = 100 µm, (B’’) = 20 µm. (C) Quantification of BrdU positive cardiomyocytes (Mef2 positive) ± S.E. A significant decrease (*p<0.05) in cardiomyocyte proliferation was detected in *Tg(hsp:dnigf1ra-GFP)* fish.

**Figure 5:**
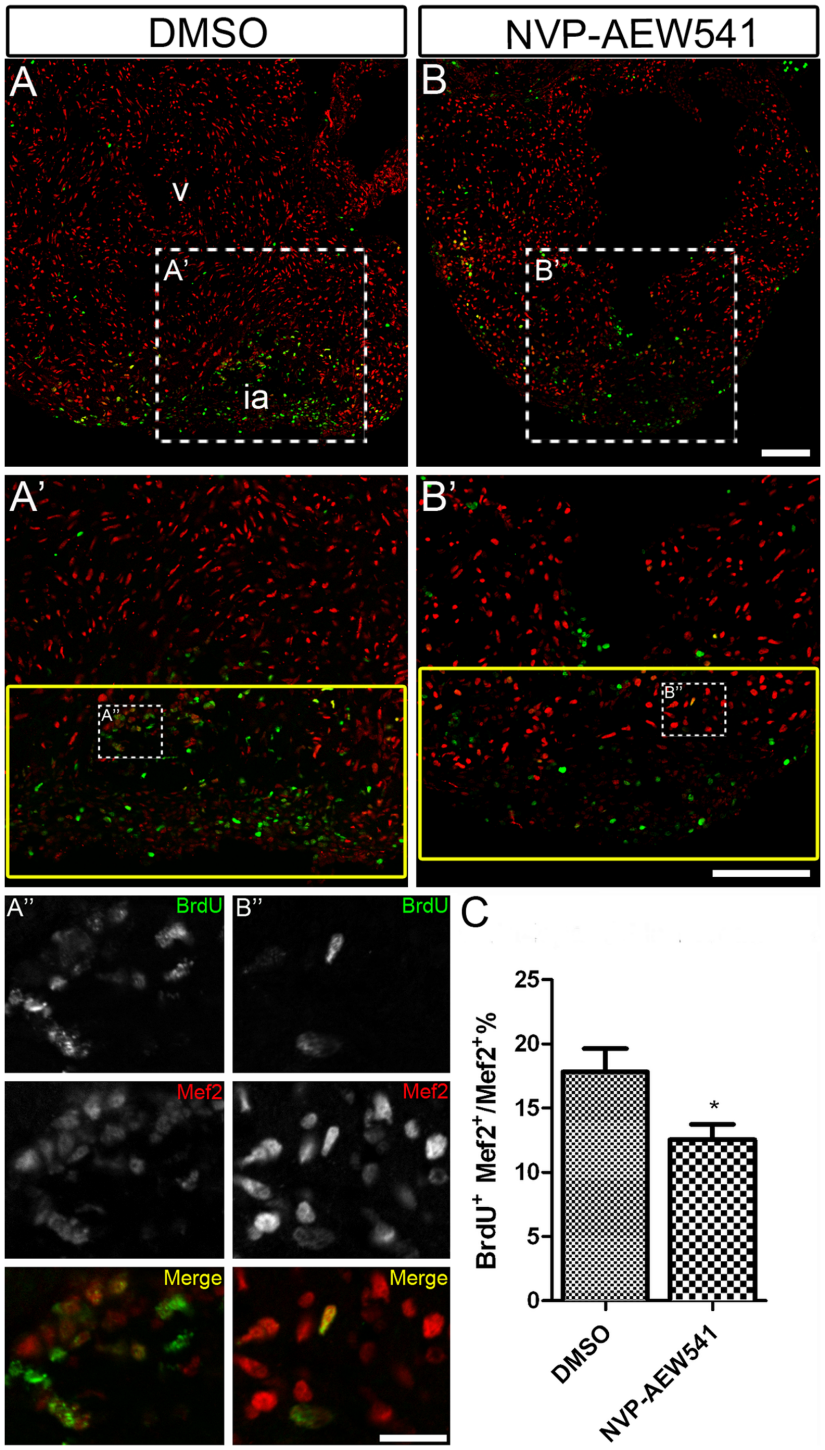
Chemical inhibition of Igf signaling suppresses cardiomyocyte proliferation during zebrafish heart regeneration. Wild type fish were treated with DMSO as a control (A, A’ and A’’) (n = 5) and the Igf1r inhibitor NVP-AEW541 (B, B’ and B’’) (n = 5) from 2–14 dpa. BrdU (green) and Mef2 (red) double positive cells indicate proliferating cardiomyocytes (A, A’, B, B’). A’ and B’ are the higher magnification images of the dashed boxes in A and B. A’’ and B’’ are the higher magnification images of the dashed boxes in A’ and B’. The yellow box indicates the wound area and cardiomyocytes were counted in this region. (A’’ and B’’), BrdU (green) and Mef2 (red) staining were shown as black and white or merged color images. Scale bar: (B, B’) = 100 µm, (B’’) = 20 µm. ia: injured area, v: ventricle. (C) Quantification of BrdU positive cardiomyocytes (Mef2 positive) ± S.E. A significant decrease (*p<0.01) in cardiomyocyte proliferation was detected in fish treated with Igf1r inhibitor NVP-AEW541.

Since *igf2b* expression is induced during zebrafish heart regeneration, we determined if Igf signaling plays a role in cardiomyocyte proliferation during regeneration, similar to its role during heart development. *Tg(hsp70:dnigf1ra-GFP)* transgenic fish and *Tg(hsp70:gal4)* control fish were heat shocked from 2–10 dpa and cardiomyocyte proliferation was measured by BrdU incorporation. Cardiomyocytes were identified by Mef2 staining. At 10 dpa, 10.08±1.14% of cardiomyocytes in the regenerating area underwent DNA synthesis in *Tg(hsp70:gal4)* control fish (Fig. 4A, A’, A’’ and C), while only 5.6±0.54% of cardiomyocytes were BrdU positive in *Tg*(*hsp70:dnigf1ra-GFP)* transgenic fish (Fig. 4B, B’, B’’ and C; see also Supplemental Fig. S5 for the non heat-shock control). Inhibition of Igf signaling significantly decreased DNA synthesis in cardiomyocyte (*p*<0.05) during heart regeneration, although inhibiting Igf signaling does not completely abolish cardiomyocyte proliferation (Fig. 4C). Similarly, a decrease in cardiomyocyte proliferation (mean ± SEM: DMSO = 17.82±1.83%; NVP-AEW541 = 12.55±1.19%) was observed in Igf1r inhibitor-treated fish (Fig. 5B, B’, B’’ and C) compared to DMSO treated control fish (Fig. 5A, A’, A’’ and C). These results suggest that Igf signaling has an important role as a mitogen in cardiomyocyte proliferation during heart regeneration.

**Figure 6:**
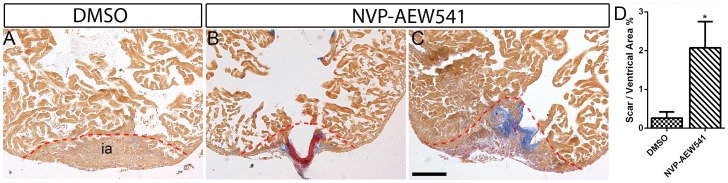
Igf signaling is required for heart regeneration. Wild type fish were treated DMSO (A) (n = 6) or the Igf1r inhibitor NVP-AEW541 (n = 7) (B and C) from 2–30 dpa. AFOG staining was performed at 30 dpa to detect collagen (blue) and fibrin (red) deposition. The dashed line marks the regenerating area. Scale bar = 100 µm. ia: injured area. (D) Quantification of scar area normalized to ventricle area in DMSO and NVP-AEW541 treated hearts. A significant increase (*p<0.05) in scar/ventricular area ratio was detected in NVP-AEW541 treated hearts.

The regenerative response of zebrafish heart to damage is thought to be able to out-compete the fibrosis and scarring responses [10], [17]. When cardiomyocyte proliferation and other responses are defective, scar tissue forms in the heart [10], [17], [33]. To determine if blocking Igf1r signaling inhibits heart regeneration, we heat shocked *Tg*(*hsp70:dnigf1ra-GFP)* fish from 2–30 dpa. Unexpectedly, we did not detect any significant increase in collagen scar formation by AFOG staining even after 30 consecutive days of heat-shock induced *dnigf1ra-GFP* transgene expression (data not shown). However, we also observed significantly decreased expression of the *dnigf1ra-GFP* transgene at 30 dpa compared to 14 dpa (Supplemental Fig. S4). To further dissect the functions of Igf signaling in heart regeneration, we treated wild type fish with NVP-AEW541 from 2–30 dpa. Majority of the DMSO treated control hearts we examined regenerated normally (Fig. 6A). By contrast, NVP-AEW541 treated hearts showed either excessive fibrin deposition (Fig. 6B) or collagen deposition (Fig. 6C). In the hearts with fibrin deposition, the myocardium appeared to be devoid of cardiomyocytes and bulged out (Fig. 6B). We quantified the amount of scar tissues as a ratio to the ventricle area and found that scar tissues are significantly increased in NVP-AEW541 treated hearts (Mean ± SEM: DMSO = 0.25±0.16%; NVP-AEW541 = 2.08±0.68%) (Fig. 6D). Collectively, these results suggest that Igf signaling is required for heart regeneration.

### Igf Signaling is Required for Contribution of*gata4:EGFP* Positive Subpopulation to the Regenerating Area

**Figure 7:**
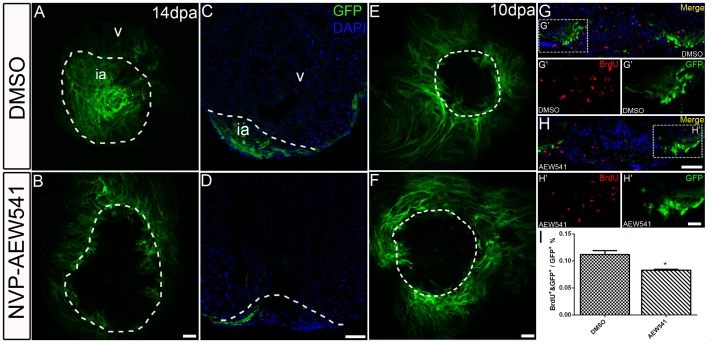
Igf signaling is required for contribution of*gata4:EGFP* positive cardiomyocytes to the regenerating area. *gata4:EGFP* fish were treated with the Igf1r inhibitor NVP-AEW541 from 2–14 dpa (n = 12) (B, D) and 7–10 dpa (n = 5) (F, H). DMSO was used as a control (14dpa: n = 10; 10 dpa: n = 6) (A, C, E, G). Whole mount confocal microscopy from the view of the apex (A, B, E, F) and frozen sections (C, D, G, H) were performed at 14 and 10 dpa to determine the contribution of the EGFP positive population. BrdU (red) and Gata4 (green) double positive cells indicate proliferating *gata4:EGFP* positive cardiomyocytes (G, G’, H, H’). G’, and H’ are the higher magnification images of the dashed boxes in G and H. BrdU staining (red) and *gata4:EGFP* (green) were shown as separated channel images. The dashed line marks the regenerating area. Scale bar: (B, D, F, H) = 50 µm; (H’) = 20 µm. ia: injured area, v: ventricle. (I) Quantification of BrdU and *gata4:EGFP* double positive cells/*gata4:EGFP* area ± S.E. A significant decrease (*p<0.05) in *gata4:EGFP* positive cell proliferation was detected in fish treated with Igf1r inhibitor NVP-AEW541 from 7–10dpa.

In a transgenic zebrafish line harboring a *gata4:EGFP* reporter, EGFP expression is activated during heart regeneration in the compact layer of myocardium [17]. This *gata4:EGFP* positive subpopulation of cardiomyocytes proliferates and contributes to the wound area to replace lost cardiomyocytes during regeneration [17]. In control hearts, *gata4:EGFP* positive cardiomyocytes became activated and covered the majority of wound area during regeneration at 14 dpa, as visualized by whole mount confocal microscopy (Fig. 7A). When we treated *Tg(gata4:EGFP)* fish with NVP-AEW541 from 2–14 dpa, we observed activation of the *gata4:EGFP* reporter, but this subpopulation of cardiomyocytes was completely excluded from the wound area (Fig. 7B). We further confirmed that blocking Igf signaling prevented *gata4:EGFP* positive cardiomyocytes from contributing to the regenerating area by imaging histological sections (Fig. 7C, D). Therefore, Igf signaling is required for the *gata4:EGFP*-positive subepicardial cardiomyocyte population to contribute to myocardial regeneration.

To further examine if Igf signaling regulates proliferation of the *gata4:EGFP* positive cardiomyocytes, we treated the *Tg(gata4:EGFP)* fish with NVP-AEW541 from 7–10 dpa when proliferation of this cardiomyocyte population occurs. After the treatment, expression of *gata4:EGFP* remain the same in DMSO treated control fish (Fig. 7E) and the NVP-AEW541 treated fish (Fig. 7F). We next examined if proliferation of *gata4:EGFP* positive cardiomyocytes was affected by BrdU incorporation. BrdU positive *gata4:EGFP* cardiomyocytes were quantified in the GFP area around the wound. Although *gata4:EGFP* cardiomyocytes still underwent DNA synthesis in NVP-AEW541 treated hearts (H, H’), we detected a significant decrease (mean ± SEM: DMSO = 0.112±0.007%; NVP-AEW541 = 0.083±0.002%) in BrdU incorporation (Fig. 7I) compared to the control (G, G). These data suggested that Igf signaling is required for proliferation of *gata4:EGFP* cardiomyocytes. However, we cannot exclude the possibility that migration of *gata4:EGFP* cardiomyocytes was also affected.

## Discussion

Cardiomyocyte proliferation plays a critical role in zebrafish myocardial regeneration [16], [17]. Growth factors, secreted molecules and extracellular matrix molecules that are essential for adult zebrafish cardiomyocyte proliferation remain to be identified. Genes and factors important for cardiomyocyte proliferation during heart development might play similar roles during regeneration. In this study, we identified Igf2 as a potential cardiomyocyte mitogen during zebrafish heart development and regeneration. Consistent with our findings, Igf2 was shown to function as a cardiomyocyte mitogen during heart growth in embryonic mouse hearts [19] and it can induce DNA synthesis of fetal rat cardiomyocytes in culture [36]. Furthermore, IGF signaling in cardiomyocytes proliferation was shown to be regulated by YAP, a transcriptional coactivator downstream in the Hippo pathway [37]. Our data further suggest that Igf signaling plays an evolutionarily conserved role regulating cardiomyocyte proliferation during heart development and might be a good candidate to promote proliferation of cardiomyocytes during repair and regeneration.

We demonstrated that Igf signaling is required for the contribution of a population of subepicardial *gata4:EGFP* positive compact myocardium to the regenerating site. Igf signaling might be required for proliferation and/or migration of this *gata4:EGFP* positive subpopulation. Whether a similar population of cardiomyocytes is present in mammalian hearts and whether it can be activated for heart repair remain to be determined. In this study, we utilized two strategies to block Igf signaling: a transgenic zebrafish line expressing a dominant negative form of Igf1ra [25], and a pharmacological inhibitor of Igf1r [35]. Using our current approaches, we cannot conclude that the effects of blocking Igf1r are cell autonomous to cardiomyocytes. Furthermore, we observed significant down-regulation and silencing of the *dnigf1ra-GFP* transgene driven by the *hsp70* promoter after 30 days of heat shock. Tissue specific knock down of Igf1r signaling in cardiomyocytes using a Cre-Lox based system will be utilized in the future to address this question, specifically in *gata4* positive cardiomyocytes. Lineage tracing will also be utilized to determine if Igf signaling regulates migration of *gata4:EGFP* cardiomyocytes. These experiments are beyond the scope of this study.

During embryonic development, the heart is initially a tube composed of endocardium and myocardium, and it grows first in an anterior-posterior direction then later concentrically by adding cardiomyocytes in a direction perpendicular to the lumen [26], [38]. Before 48 hpf, relative little proliferation can be detected in cardiomyocytes and the heart grows by distinct phases of cardiomyocyte differentiation [32]. Myocardial growth and wall thickening starts in mid gestation in mouse embryos and epicardium is a potential source of mitogens for cardiomyocytes [39]. In zebrafish embryos, the proepicardial organ is first visible at 48 hpf and starts to cover the myocardium at 72 hpf [40]. The timing of cardiomyocyte proliferation we observed correlates well with the timing of when the epicardium appears and starts to cover the myocardium [40]. Thus, the epicardium might be also a source of mitogens for cardiomyocytes in zebrafish embryos. However, we cannot rule out that the endocardium is another source of mitogens for cardiomyocyte proliferation in zebrafish.

Our data suggest that Igf signaling is required for zebrafish heart regeneration. Nonetheless, Igf2 is probably not the only cardiomyocyte mitogen that is utilized during regeneration; other growth factors likely account for the remaining proliferative capacity that supports regeneration. Tgfß/Activin signaling plays important roles in cardiomyocyte proliferation and scar formation [41], [42]. Hedgehog signaling was also recently shown to be important for cardiomyocyte proliferation during heart development and regeneration [42]. Furthermore, the Hippo-YAP pathway regulates cardiomyocyte proliferation and heart size by coupling to IGF signaling in mice [37]. It is possible that Igf signaling coordinates with these signaling pathways to regulate cardiomyocyte proliferation during zebrafish heart regeneration. Furthermore, TGFß/Activin signaling might be involved in the beneficial interplay between scar-based repair and cardiomyocyte proliferation based regeneration during heart regeneration [41]. This dynamic interaction likely depends on the balance between scar formation and myocardial regeneration, and loss of Igf signaling may shift the balance towards scar formation.

## Supporting Information

Figure S1 ***igf2a*** **expression is not detected during zebrafish heart regeneration.** ISH was performed on uncut hearts (A) and 3 dpa (B), 7 dpa (C), 10 dpa (D) and 14 dpa (E) regenerating hearts. There is no detectable *igf2a* expression in regenerating hearts by ISH. Scale bar = 100 µm. v: ventricle; ia: injured area. It was shown previously that embryos injected with *igf1r* morpholinos exhibited severely reduced body length at 24 hpf [43], raising the possibility that blocking Igf signaling inhibits overall embryonic growth and development in general. We examined embryo length and overall development of *Tg*(*hsp70:dnigf1ra-GFP*) embryos or embryos treated with the Igf1r chemical inhibitor NVP-AEW-541 from 48–72 hpf. We observed only a very mild reduction in the length of the inhibitor treated (3.7%) and transgenic embryos (5.7%) (Figure S2. A–E). These results suggest that the reduced cardiomyocyte numbers are unlikely caused by effects of Igf signaling on overall embryo growth and development indicating Igf signaling is required for zebrafish heart development.
(TIF)

Figure S2 **Inhibiting Igf signaling in zebrafish embryos results in mild defects in overall length.** WT embryos were treated with DMSO as a control (A) (n = 20) or the Igf inhibitor NVP-AEW541 (B) (n = 20) from 48–72 hpf. *Tg(hsp70:dnigf1ra-GFP)* (D and E) and non-transgenic control (C) embryos were heat shocked twice at 48 and 72 hpf for 30 mins at 40°C. The embryos were observed at 76 hpf using a confocal microscope. *Tg(hsp70:dnigf1ra-GFP)* were identified by GFP expression. The length of the inhibitor treated embryos is about 3.2% shorter than untreated control embryos, and the *Tg(hsp70:dnigf1ra-GFP)* transgenic embryos are 5.7% % shorter than non-transgenic control embryos. Scale bar: (B) = 200 µm.
(TIF)

Figure S3 **Inhibiting Igf signaling in zebrafish embryos results in abnormal cardiac looping.** *Tg(myl7:GFP)* embryos were treated with DMSO as a control (A) (n = 20) or the Igf inhibitor NVP-AEW541 (B) (n = 20) from 48–72 hpf. The embryos were observed at 76 hpf using a fluorescence microscope. Scale bar = 20 µm.
(TIF)

Figure S4 **Down regulation of the** ***dnigf1ra*** **transgene is detected after 30 days of heat shock.** Wild type or *Tg(hsp70:dnigf1ra-GFP)* were heat shocked from 2–14 or 2–30 dpa. ISH was performed to assess *dnigf1ra* transgene expression in 14 dpa and 30 dpa regenerating hearts. Strong *dnigf1ra* transgene expression was detected at 14 dpa (B). Weak or no expression of the *dnigf1ra* transgene was detected at 30 dpa (D). Scale bar = 100 µm. v: ventricle, ia: injured area, epi: epicardium.
(TIF)

Figure S5 **Non heat-shocked control for the** ***Tg(hsp70:dnigf1ra-GFP)*** **fish experiment.** BrdU incorporation was determined in non heat-shocked (Non-HS) *Tg(hsp70:dnigf1ra-GFP)* control fish (n = 4) (A and A’) and heat shocked (HS) *Tg(hsp70:dnigf1ra-GFP)* transgenic zebrafish (n = 6) (B and B’). *Tg(hsp70:dnigf1ra-GFP)* transgenic were heat shocked for 1 h at 38°C after amputation from 2–10 dpa. Non heat-shocked control fish were kept in the regular system. BrdU (green) and Mef2 (red) double positive cells indicate proliferating cardiomyocytes (A, A’, B, B’). A’ and B’ are the higher magnification images of the dashed boxes in A and B. The yellow box indicates the wound area; cardiomyocytes were counted in this region. BrdU (green) and Mef2 (red) staining were shown single channeled or merged color images. ia: injured area, v: ventricle. Scale Bar = 20 µm. (C) Quantification of BrdU positive cardiomyocytes (Mef2 positive) ± S.E. A significant decrease (*p<0.05) in cardiomyocyte proliferation was detected in *Tg(hsp70:dnigf1ra-GFP)* fish.
(TIF)

## References

[1] Li F, Wang X, Capasso JM, Gerdes AM (1996) Rapid transition of cardiac myocytes from hyperplasia to hypertrophy during postnatal development. J Mol Cell Cardiol 28: 1737–1746. 8877783 10.1006/jmcc.1996.0163

[2] Pasumarthi KB, Field LJ (2002) Cardiomyocyte cell cycle regulation. Circ Res 90: 1044–1054. 12039793 10.1161/01.res.0000020201.44772.67

[3] van Amerongen MJ, Engel FB (2008) Features of cardiomyocyte proliferation and its potential for cardiac regeneration. J Cell Mol Med 12: 2233–2244. 18662194 10.1111/j.1582-4934.2008.00439.x PMC4514102

[4] Walsh S, Ponten A, Fleischmann BK, Jovinge S (2010) Cardiomyocyte cell cycle control and growth estimation in vivo–an analysis based on cardiomyocyte nuclei. Cardiovasc Res 86: 365–373. 20071355 10.1093/cvr/cvq005

[5] Roger VL, Go AS, Lloyd-Jones DM, Benjamin EJ, Berry JD, et al (2012) Heart disease and stroke statistics–2012 update: a report from the American Heart Association. Circulation 125: e2–e220. 22179539 10.1161/CIR.0b013e31823ac046 PMC4440543

[6] Beltrami AP, Urbanek K, Kajstura J, Yan SM, Finato N, et al (2001) Evidence that human cardiac myocytes divide after myocardial infarction. N Engl J Med 344: 1750–1757. 11396441 10.1056/NEJM200106073442303

[7] Bergmann O, Bhardwaj RD, Bernard S, Zdunek S, Barnabe-Heider F, et al (2009) Evidence for cardiomyocyte renewal in humans. Science 324: 98–102. 19342590 10.1126/science.1164680 PMC2991140

[8] Bersell K, Arab S, Haring B, Kuhn B (2009) Neuregulin1/ErbB4 signaling induces cardiomyocyte proliferation and repair of heart injury. Cell 138: 257–270. 19632177 10.1016/j.cell.2009.04.060

[9] Engel FB, Hsieh PC, Lee RT, Keating MT (2006) FGF1/p38 MAP kinase inhibitor therapy induces cardiomyocyte mitosis, reduces scarring, and rescues function after myocardial infarction. Proc Natl Acad Sci U S A 103: 15546–15551. 17032753 10.1073/pnas.0607382103 PMC1622860

[10] Poss KD, Wilson LG, Keating MT (2002) Heart regeneration in zebrafish. Science 298: 2188–2190. 12481136 10.1126/science.1077857

[11] Raya A, Koth CM, Buscher D, Kawakami Y, Itoh T, et al (2003) Activation of Notch signaling pathway precedes heart regeneration in zebrafish. Proc Natl Acad Sci U S A 100 Suppl 111889–11895. 12909711 10.1073/pnas.1834204100 PMC304103

[12] Poss KD (2007) Getting to the heart of regeneration in zebrafish. Semin Cell Dev Biol 18: 36–45. 17178459 10.1016/j.semcdb.2006.11.009

[13] Choi WY, Poss KD (2012) Cardiac regeneration. Curr Top Dev Biol 100: 319–344. 22449849 10.1016/B978-0-12-387786-4.00010-5 PMC3342383

[14] Lien CL, Harrison MR, Tuan TL, Starnes VA (2012) Heart repair and regeneration: Recent insights from zebrafish studies. Wound Repair Regen 20: 638–646. 22818295 10.1111/j.1524-475X.2012.00814.x PMC3445789

[15] Kikuchi K, Poss KD (2012) Cardiac regenerative capacity and mechanisms. Annu Rev Cell Dev Biol 28: 719–741. 23057748 10.1146/annurev-cellbio-101011-155739 PMC3586268

[16] Jopling C, Sleep E, Raya M, Marti M, Raya A, et al (2010) Zebrafish heart regeneration occurs by cardiomyocyte dedifferentiation and proliferation. Nature 464: 606–609. 20336145 10.1038/nature08899 PMC2846535

[17] Kikuchi K, Holdway JE, Werdich AA, Anderson RM, Fang Y, et al (2010) Primary contribution to zebrafish heart regeneration by gata4(+) cardiomyocytes. Nature 464: 601–605. 20336144 10.1038/nature08804 PMC3040215

[18] Brade T, Kumar S, Cunningham TJ, Chatzi C, Zhao X, et al (2011) Retinoic acid stimulates myocardial expansion by induction of hepatic erythropoietin which activates epicardial Igf2. Development 138: 139–148. 21138976 10.1242/dev.054239 PMC2998168

[19] Li P, Cavallero S, Gu Y, Chen TH, Hughes J, et al (2011) IGF signaling directs ventricular cardiomyocyte proliferation during embryonic heart development. Development 138: 1795–1805. 21429986 10.1242/dev.054338 PMC3074453

[20] Chao W, D'Amore PA (2008) IGF2: epigenetic regulation and role in development and disease. Cytokine Growth Factor Rev 19: 111–120. 18308616 10.1016/j.cytogfr.2008.01.005 PMC2314671

[21] LeRoith D (2008) Clinical relevance of systemic and local IGF-I: lessons from animal models. Pediatr Endocrinol Rev 5 Suppl 2739–743. 18317445

[22] Maures T, Chan SJ, Xu B, Sun H, Ding J, et al (2002) Structural, biochemical, and expression analysis of two distinct insulin-like growth factor I receptors and their ligands in zebrafish. Endocrinology 143: 1858–1871. 11956169 10.1210/endo.143.5.8768

[23] Chablais F, Jazwinska A (2010) IGF signaling between blastema and wound epidermis is required for fin regeneration. Development 137: 871–879. 20179093 10.1242/dev.043885

[24] Nusslein-Volhard CD, R (2002) Zebrafish, A Practical Approach. New York, USA.

[25] Kamei H, Ding Y, Kajimura S, Wells M, Chiang P, et al (2011) Role of IGF signaling in catch-up growth and accelerated temporal development in zebrafish embryos in response to oxygen availability. Development 138: 777–786. 21266413 10.1242/dev.056853

[26] Mably JD, Mohideen MA, Burns CG, Chen JN, Fishman MC (2003) heart of glass regulates the concentric growth of the heart in zebrafish. Curr Biol 13: 2138–2147. 14680629 10.1016/j.cub.2003.11.055

[27] Burns CG, Milan DJ, Grande EJ, Rottbauer W, MacRae CA, et al (2005) High-throughput assay for small molecules that modulate zebrafish embryonic heart rate. Nat Chem Biol 1: 263–264. 16408054 10.1038/nchembio732

[28] Heicklen-Klein A, Evans T (2004) T-box binding sites are required for activity of a cardiac GATA-4 enhancer. Dev Biol 267: 490–504. 15013808 10.1016/j.ydbio.2003.09.042

[29] Kim J, Rubin N, Huang Y, Tuan TL, Lien CL (2012) In vitro culture of epicardial cells from adult zebrafish heart on a fibrin matrix. Nat Protoc 7: 247–255. 22262006 10.1038/nprot.2011.440 PMC3541820

[30] Kim J, Wu Q, Zhang Y, Wiens KM, Huang Y, et al (2010) PDGF signaling is required for epicardial function and blood vessel formation in regenerating zebrafish hearts. Proc Natl Acad Sci U S A 107: 17206–17210. 20858732 10.1073/pnas.0915016107 PMC2951463

[31] Lien CL, Schebesta M, Makino S, Weber GJ, Keating MT (2006) Gene expression analysis of zebrafish heart regeneration. PLoS Biol 4: e260. 16869712 10.1371/journal.pbio.0040260 PMC1523227

[32] de Pater E, Clijsters L, Marques SR, Lin YF, Garavito-Aguilar ZV, et al (2009) Distinct phases of cardiomyocyte differentiation regulate growth of the zebrafish heart. Development 136: 1633–1641. 19395641 10.1242/dev.030924 PMC2673760

[33] Lepilina A, Coon AN, Kikuchi K, Holdway JE, Roberts RW, et al (2006) A dynamic epicardial injury response supports progenitor cell activity during zebrafish heart regeneration. Cell 127: 607–619. 17081981 10.1016/j.cell.2006.08.052

[34] Hartnett L, Glynn C, Nolan CM, Grealy M, Byrnes L (2010) Insulin-like growth factor-2 regulates early neural and cardiovascular system development in zebrafish embryos. Int J Dev Biol 54: 573–583. 19757379 10.1387/ijdb.092922lh

[35] Garcia-Echeverria C, Pearson MA, Marti A, Meyer T, Mestan J, et al (2004) In vivo antitumor activity of NVP-AEW541-A novel, potent, and selective inhibitor of the IGF-IR kinase. Cancer Cell 5: 231–239. 15050915 10.1016/s1535-6108(04)00051-0

[36] Liu Q, Yan H, Dawes NJ, Mottino GA, Frank JS, et al (1996) Insulin-like growth factor II induces DNA synthesis in fetal ventricular myocytes in vitro. Circ Res 79: 716–726. 8831495 10.1161/01.res.79.4.716

[37] Xin M, Kim Y, Sutherland LB, Qi X, McAnally J, et al (2011) Regulation of insulin-like growth factor signaling by Yap governs cardiomyocyte proliferation and embryonic heart size. Sci Signal 4: ra70. 22028467 10.1126/scisignal.2002278 PMC3440872

[38] Mably JD, Chuang LP, Serluca FC, Mohideen MA, Chen JN, et al (2006) santa and valentine pattern concentric growth of cardiac myocardium in the zebrafish. Development 133: 3139–3146. 16873582 10.1242/dev.02469

[39] Sucov HM, Gu Y, Thomas S, Li P, Pashmforoush M (2009) Epicardial control of myocardial proliferation and morphogenesis. Pediatr Cardiol 30: 617–625. 19277768 10.1007/s00246-009-9391-8

[40] Serluca FC (2008) Development of the proepicardial organ in the zebrafish. Dev Biol 315: 18–27. 18206866 10.1016/j.ydbio.2007.10.007

[41] Chablais F, Jazwinska A (2012) The regenerative capacity of the zebrafish heart is dependent on TGFbeta signaling. Development 139: 1921–1930. 22513374 10.1242/dev.078543

[42] Choi WY, Gemberling M, Wang J, Holdway JE, Shen MC, et al (2013) In vivo monitoring of cardiomyocyte proliferation to identify chemical modifiers of heart regeneration. Development 140: 660–666. 23293297 10.1242/dev.088526 PMC3561784

[43] Schlueter PJ, Peng G, Westerfield M, Duan C (2007) Insulin-like growth factor signaling regulates zebrafish embryonic growth and development by promoting cell survival and cell cycle progression. Cell Death Differ 14: 1095–1105. 17332774 10.1038/sj.cdd.4402109

