# Correction: Igf Signaling is Required for Cardiomyocyte Proliferation during Zebrafish Heart Development and Regeneration

**DOI:** 10.1371/annotation/f009e61f-85e8-4709-a1ed-1dc2e649f8a3

**Published:** 2014-01-17

**Authors:** Ying Huang, Michael R. Harrison, Arthela Osorio, Jieun Kim, Aaron Baugh, Cunming Duan, Henry M. Sucov, Ching-Ling Lien

Dr. Hua Shen was not included in the author byline. Dr. Shen should be listed as the sixth author and affiliated with institution number 5. The contributions of this author are as follows: Performed the experiments.

