# Supplementary figures and images for: Igf Signaling is Required for Cardiomyocyte Proliferation during Zebrafish Heart Development and Regeneration
Source: PLoS One. 2013 Jun 26;8(6):e67266. doi: 10.1371/journal.pone.0067266 (PMC3694143; doi:10.1371/journal.pone.0067266)

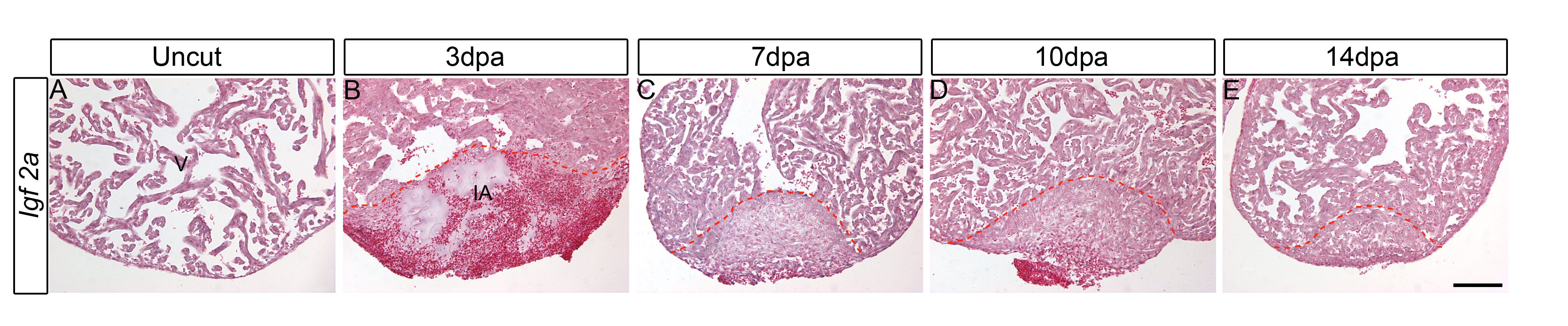

Supplement: Figure S1 — igf2a expression is not detected during zebrafish heart regeneration. ISH was performed on uncut hearts (A) and 3 dpa (B), 7 dpa (C), 10 dpa (D) and 14 dpa (E) regenerating hearts. There is no detectable igf2a expression in regenerating hearts by ISH. Scale bar = 100 µm. v: ventricle; ia: injured area. It was shown previously that embryos injected with igf1r morpholinos exhibited severely reduced body length at 24 hpf [43], raising the possibility that blocking Igf signaling inhibits overall embryonic growth and development in general. We examined embryo length and overall development of Tg(hsp70:dnigf1ra-GFP) embryos or embryos treated with the Igf1r chemical inhibitor NVP-AEW-541 from 48–72 hpf. We observed only a very mild reduction in the length of the inhibitor treated (3.7%) and transgenic embryos (5.7%) (Figure S2. A–E). These results suggest that the reduced cardiomyocyte numbers are unlikely caused by effects of Igf signaling on overall embryo growth and development indicating Igf signaling is required for zebrafish heart development. (TIF) [file pone.0067266.s001.tif]

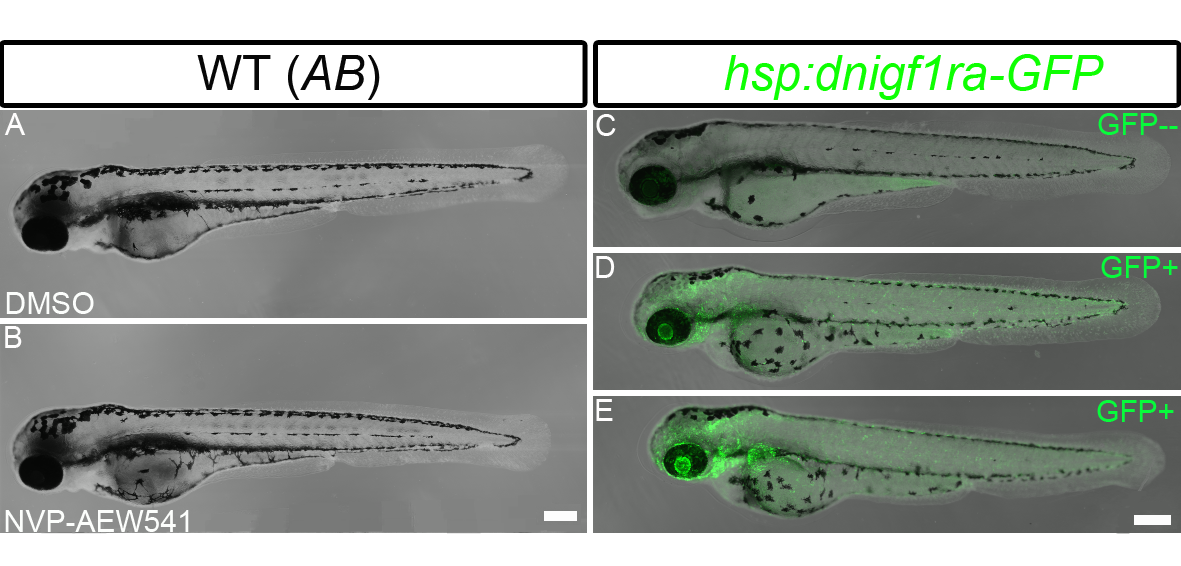

Supplement: Figure S2 — Inhibiting Igf signaling in zebrafish embryos results in mild defects in overall length. WT embryos were treated with DMSO as a control (A) (n = 20) or the Igf inhibitor NVP-AEW541 (B) (n = 20) from 48–72 hpf. Tg(hsp70:dnigf1ra-GFP) (D and E) and non-transgenic control (C) embryos were heat shocked twice at 48 and 72 hpf for 30 mins at 40°C. The embryos were observed at 76 hpf using a confocal microscope. Tg(hsp70:dnigf1ra-GFP) were identified by GFP expression. The length of the inhibitor treated embryos is about 3.2% shorter than untreated control embryos, and the Tg(hsp70:dnigf1ra-GFP) transgenic embryos are 5.7% % shorter than non-transgenic control embryos. Scale bar: (B) = 200 µm. (TIF) [file pone.0067266.s002.tif]

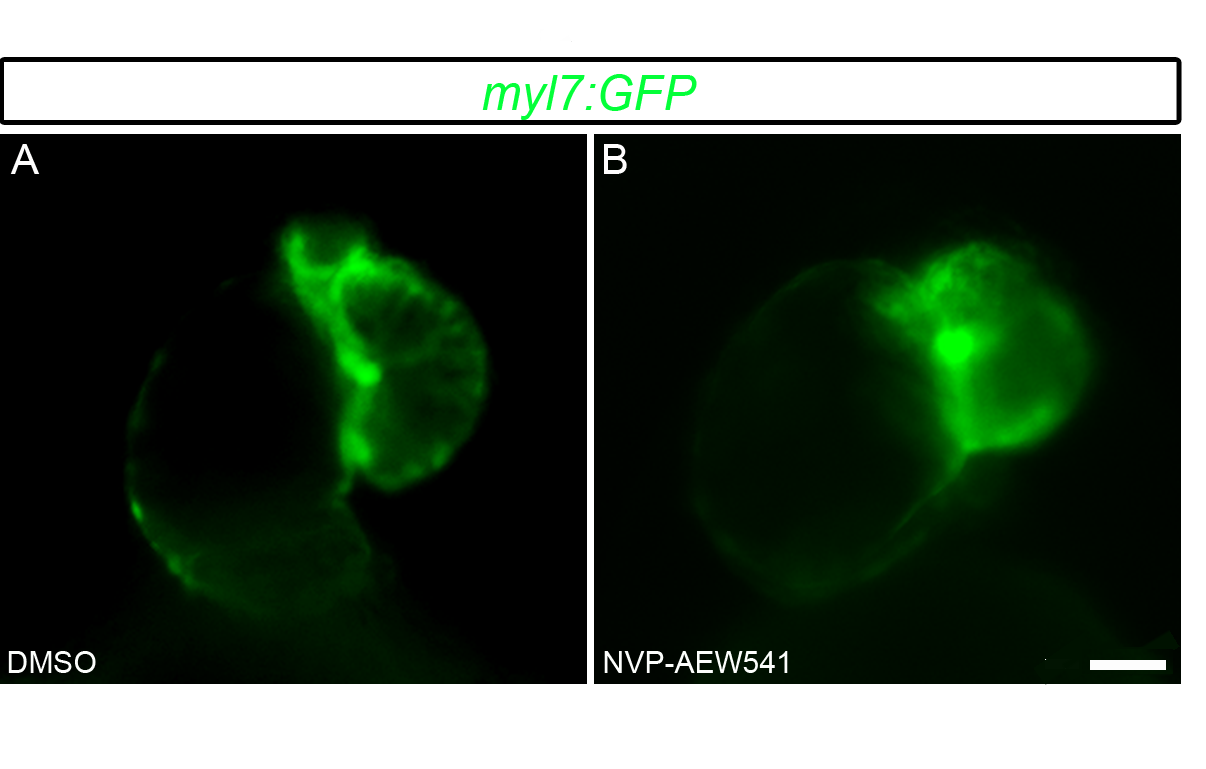

Supplement: Figure S3 — Inhibiting Igf signaling in zebrafish embryos results in abnormal cardiac looping. Tg(myl7:GFP) embryos were treated with DMSO as a control (A) (n = 20) or the Igf inhibitor NVP-AEW541 (B) (n = 20) from 48–72 hpf. The embryos were observed at 76 hpf using a fluorescence microscope. Scale bar = 20 µm. (TIF) [file pone.0067266.s003.tif]

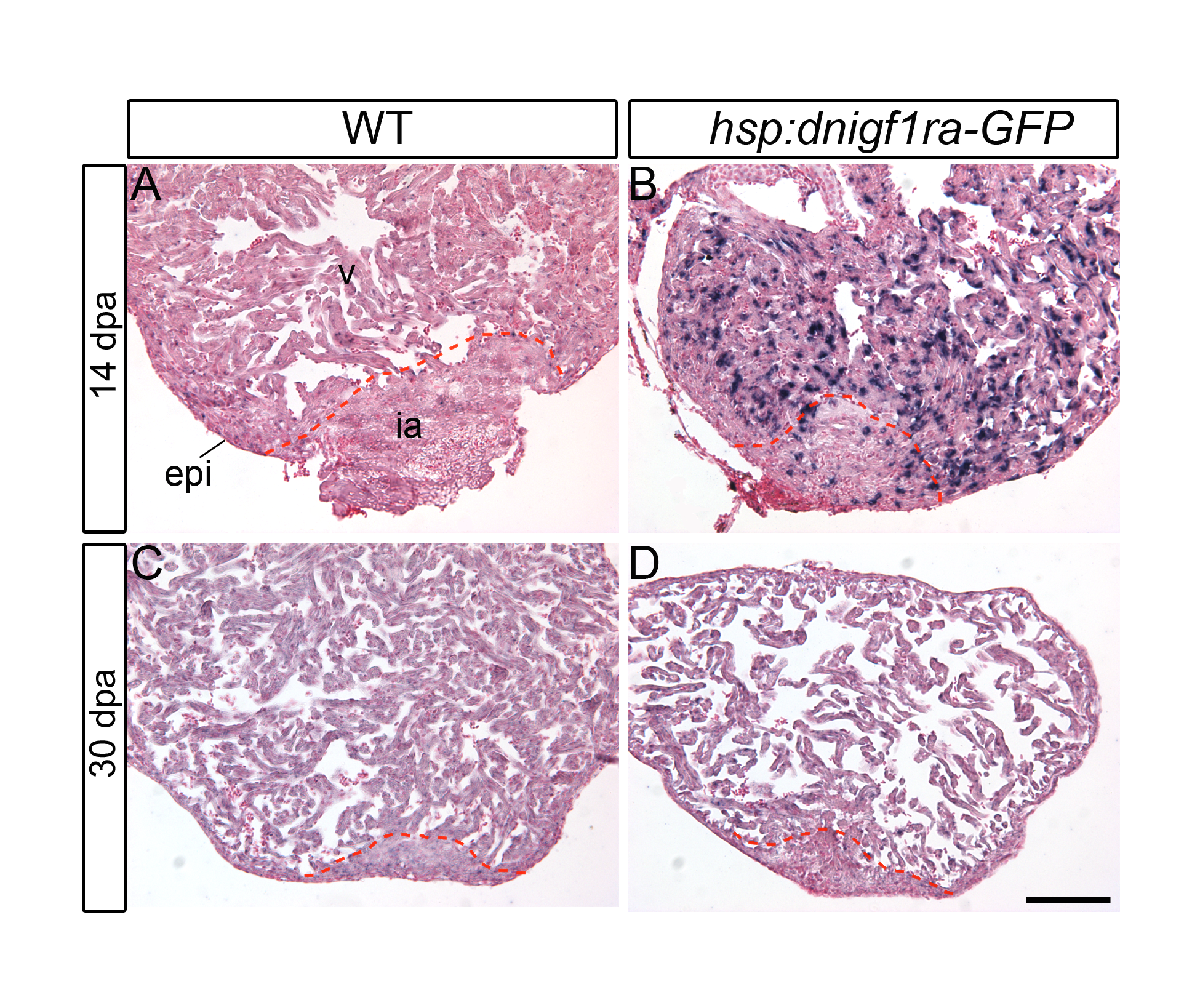

Supplement: Figure S4 — Down regulation of the dnigf1ra transgene is detected after 30 days of heat shock. Wild type or Tg(hsp70:dnigf1ra-GFP) were heat shocked from 2–14 or 2–30 dpa. ISH was performed to assess dnigf1ra transgene expression in 14 dpa and 30 dpa regenerating hearts. Strong dnigf1ra transgene expression was detected at 14 dpa (B). Weak or no expression of the dnigf1ra transgene was detected at 30 dpa (D). Scale bar = 100 µm. v: ventricle, ia: injured area, epi: epicardium. (TIF) [file pone.0067266.s004.tif]

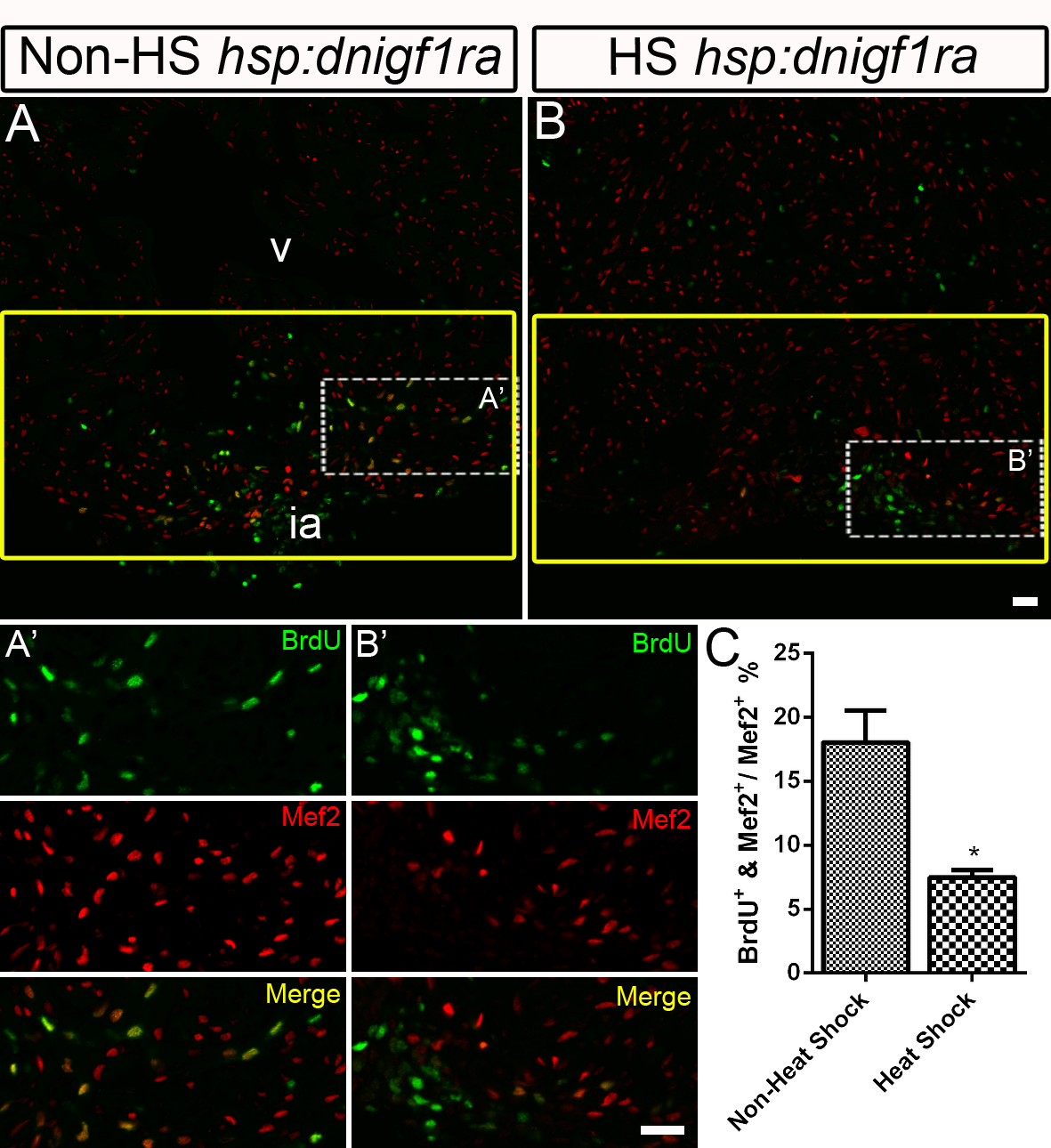

Supplement: Figure S5 — Non heat-shocked control for the Tg(hsp70:dnigf1ra-GFP) fish experiment. BrdU incorporation was determined in non heat-shocked (Non-HS) Tg(hsp70:dnigf1ra-GFP) control fish (n = 4) (A and A’) and heat shocked (HS) Tg(hsp70:dnigf1ra-GFP) transgenic zebrafish (n = 6) (B and B’). Tg(hsp70:dnigf1ra-GFP) transgenic were heat shocked for 1 h at 38°C after amputation from 2–10 dpa. Non heat-shocked control fish were kept in the regular system. BrdU (green) and Mef2 (red) double positive cells indicate proliferating cardiomyocytes (A, A’, B, B’). A’ and B’ are the higher magnification images of the dashed boxes in A and B. The yellow box indicates the wound area; cardiomyocytes were counted in this region. BrdU (green) and Mef2 (red) staining were shown single channeled or merged color images. ia: injured area, v: ventricle. Scale Bar = 20 µm. (C) Quantification of BrdU positive cardiomyocytes (Mef2 positive) ± S.E. A significant decrease (*p<0.05) in cardiomyocyte proliferation was detected in Tg(hsp70:dnigf1ra-GFP) fish. (TIF) [file pone.0067266.s005.tif]
